# Blood meal analysis: host-feeding patterns of biting midges (Diptera, Ceratopogonidae, *Culicoides* Latreille) in Slovakia

**DOI:** 10.1051/parasite/2021058

**Published:** 2021-07-20

**Authors:** Zuzana Kasičová, Andrea Schreiberová, Andrea Kimáková, Alica Kočišová

**Affiliations:** Department of Epizootiology, Parasitology and Protection of One Health, University of Veterinary Medicine and Pharmacy in Košice Komenského 73 041 81 Košice Slovak Republic

**Keywords:** *Culicoides*, Vector identification, Species-specific PCR analysis, Slovakia

## Abstract

Biting midges of the genus *Culicoides* are vectors of important pathogens affecting domestic and wild animals and have played a major role in the re-emergence of new outbreaks of bluetongue (BTV) and Schmallenberg (SBV) viruses in Europe. To determine vector-host specificity, trophic preference from blood meal analysis is of major importance in the surveillance of arthropod-borne diseases. Of 28,752 specimens collected, we identified 17 *Culicoides* species and investigated a total of 48 host sequences from the blood meals. *Culicoides obsoletus/C. scoticus*, *C. dewulfi*, *C. pulicaris*, *C. lupicaris*, *C. punctatus*, *C. newsteadi*, *C. riethi*, and *C. furcillatus* were found to feed on mammals (cattle, horses, and humans), birds (domestic chickens), small rodents (*Apodemus flavicollis*), and hares (*Lepus europaeus*). To our knowledge, this is the first study investigating trophic preferences of *Culicoides* spp. in Slovakia. This study demonstrated that *Culicoides* species are able to feed on domesticated host vertebrates as well as birds, rodents, and humans.

## Abstract (fr)

Les moucherons piqueurs du genre *Culicoides* sont des vecteurs d’agents pathogènes importants affectant les animaux domestiques et sauvages et ont joué un rôle majeur dans la réémergence de nouvelles épidémies de la fièvre catarrhale ovine (FCO) et de la maladie à virus Schmallenberg (SBV) en Europe. Pour déterminer la spécificité vecteur-hôte, la préférence trophique issue de l’analyse des repas sanguins est d’une importance majeure dans la surveillance des maladies transmises par les arthropodes. À partir de 28 752 spécimens collectés, nous avons identifié 17 espèces de *Culicoides* et étudié un total de 48 séquences d’hôtes à partir des repas de sang. Nous avons trouvé que *Culicoides obsoletus* / *C. scoticus*, *C. dewulfi*, *C. pulicaris*, *C. lupicaris*, *C. punctatus*, *C. newsteadi*, *C. riethi* et *C. furcillatus* se nourrissent à partir de mammifères (bovins, chevaux et humains), oiseaux (poulets domestiques), petits rongeurs (*Apodemus flavicollis*) et lièvres (*Lepus europaeus*). À notre connaissance, il s’agit de la première étude portant sur les préférences trophiques de *Culicoides* spp. en Slovaquie. Cette étude a démontré que des espèces de *Culicoides* sont capables de se nourrir d’hôtes vertébrés domestiqués ainsi que d’oiseaux, de rongeurs et d’humains.

## Introduction

The biting midges of the genus *Culicoides* (Diptera: Ceratopogonidae) are small hematophagous Diptera which are important for their ability to transmit pathogens of microbial and parasitic origin. Only females are hematophagous and they use blood as a source of proteins necessary for laying eggs. So far, 1347 species of biting midges have been described in the world [10]; of these, 64 species have been confirmed in Slovakia [41, 43]. After 2006, when bluetongue virus serotype 8 began to spread extensively, there was increased interest in monitoring biting midge occurrence and in investigating species composition [50]. A few years later, the Schmallenberg virus caused significant economic losses as it spread from Germany [5, 44] to other countries, including Slovakia [40]. From the epizootiological point of view, with regard to the spread of these diseases and recurrence of new foci of infections in Europe, in particular in Germany, Belgium, and France [11, 19, 20], it was necessary to monitor the correlations between the causal agents, vectors, and susceptible hosts. The most important vectors of these viruses in the Palaearctic region were *Culicoides obsoletus*/*C. scoticus*, which were also confirmed as the most frequent species by Sarvašová et al. in previous investigations in Eastern Slovakia [41]. The research performed so far in Slovakia has been focussed on monitoring faunistic data, but the host preferences have not yet been studied. One of the first steps within the efforts aimed at understanding the relationships between parasites and hosts was to identify the hosts from which biting midges take blood. Ceratopogonids are vectors of parasites and pathogens between their vertebrate hosts. Little information is available on the circadian Palaearctic biting midges. The biting cycle follows a circadian rhythm. In general, the prevailing view is that midges are most active at dusk. Species of *Leptoconops* and *Lasiohelea* are diurnal, as are a small number of *Culicoides* (e.g. *C. impunctatus*, *C. obsoletus* or *C. nubeculosus*) [3], but most *Culicoides* are crepuscular and/or nocturnal. Diurnal species commonly show two peaks of activity, in the morning (2–3 h after sunrise) and in the afternoon (close to sunset). According to our previous research [42], the highest flight activity of *C. obsoletus*/*C. scoticus* and *C. punctatus* was observed between midnight and 2.00 am, and of *C. pulicaris* and *C. newsteadi* between 2.00 and 4.00 am. A similar survey found that *C. punctatus* activity increased from 8 pm and peaked between 2.00 and 4.00 am in Turkey [14]. Environmental conditions may modify basic activity patterns. The frequency of feeding varies with species and situation [25, 38]; host availability plays an important role in the feeding behavior of biting midges in general. Most ceratopogonids are mammalophilic or ornithophilic, although some feed on reptiles [9]. Molecular identification of the vertebrate hosts of species of ceratopogonids in Europe has been conducted for 31 species that suck blood on 45 different hosts. Thirty-three species of birds and 12 species of mammals have been identified as hosts [27]. Determining the transmission dynamics of biting midges and pathogens requires an understanding of the interactions between these midges and vertebrate host communities. Such interactions are characterized through molecular methods that determine the taxonomic origin of a ceratopogonid blood meal. Several polymerase chain reaction (PCR) assays have been developed to determine host blood of midges, and these assays can be done based on several genes. The COI (*cytochrome c oxidase subunit I*), Cyt b (*cytochrome b*) [36, 46] and 16 S rRNA (16S ribosomal RNA) genes are used for blood meal analysis of vertebrates [45]. The purpose of our study was to examine the host’s blood in biting midges captured on cattle and horse farms. This was the first study in Slovakia to analyse the detected host’s blood based on a segment of the mitochondrial gene (*cytochrome b*).

## Materials and methods

### Study sites and trapping procedure

Biting midges were captured in 2019 on cattle farms located near villages in Eastern Slovakia, in particular: Turňa nad Bodvou (GPS: 48.6004045, 20.8776599), Ostrov (GPS: 48.7424000, 21.2467712) Horňa (GPS: 48.757778, 22.201944), and near horses kept in a horse-riding arena in Košice (GPS: 48.7163857, 21.2610746). Midges were collected using CDC miniature UV light traps, model 1212 (John W. Hock Company, Gainesville, FL, USA), each fitted with black fluorescent (ultraviolet) light tubes and a 6 V battery. The traps were operated between feedlots, ensuring proximity between the trap and the potential host, at two-week intervals from April to November 2019. The traps were installed in the evening, approximately 1 h before dusk, and collected on the next day, early in the morning, approximately 2 h after sunrise. Sixteen individual collections (trapping nights) were made at each study site. Biting midges were captured together with other insects in a container with approximately 250 mL of 50% ethanol.

### Laboratory processing of biting midges and their morphological diagnostics

Biting midges were diagnosed using the morphological keys of Mathieu et al. [28]; based on their characteristic wing spots, they were divided into individual complexes, and individual species were identified under a binocular microscope (Zeiss Stemi DV4). Biting midges were stored in 70% ethanol and kept in a refrigerator at +4 °C. The physiological condition of females was identified according to Dyce [15], based on burgundy pigment present in females’ abdomens. Four groups of biting midges were differentiated: nulliparous (empty abdomen without the presence of blood, i.e., they never sucked blood); parous (empty abdomen, with traces of burgundy pigment after blood sucking); engorged (abdomen filled with blood); and gravid (abdomen with eggs). Females with abdomen filled with blood were subjected to PCR analysis of the host’s blood.

### DNA extraction

The total genomic DNA of blood-engorged *Culicoides* females was extracted using a DNeasy^®^ Blood and Tissue Kit (QIAGEN, GmbH, Hilden, Germany), following the manufacturer’s protocol. To obtain DNA from female midges, we used the whole body, which we removed from residual water in 96% ethanol before extraction. The concentrations of extracted DNA were measured by a Nano Drop Lite Spectrophotometer (Thermo Scientific, USA) and diluted to the final concentration of 20–50 ng/μL. The samples were stored in a freezer at −20 °C and used for the PCR assay.

### Identification of *Culicoides* species

The molecular identification of *Culicoides* spp. was based on the sequencing of partial mitochondrial cytochrome c oxidase subunit I gene (COI) sequences representing an identification barcode for the species. *Culicoides obsoletus*/*Culicoides scoticus* is determined only based on morphological features (Table 1). By repeated PCR analysis, we could not determine the *Culicoides* species. The partial COI sequences for the individual midges were obtained using forward C1-J-1718 (5′–GGAGGATTTGGAAATTGATTAGT–3′) and reverse C1-N-2191 (5′–CAGGTAAAATTAAAATATAAACTTCTGG–3′) primers [10, 13]. The resulting length of the amplified COI fragments was about 523 bp.

**Table 1:** Host blood meals from blood-fed *Culicoides* spp.

Sample Number (Trap site)	Species after identification in NCBI	GenBank	*Bos taurus*	*Equus caballus*	*Gallus gallus*	*Apodemus flavicollis*	*Lepus europaeus*	*Homo sapiens*	Not detected
**Košice**									
Csp1	*C. obsoletus*	MW633115	–	MW657642	–	–	–	–	–
Csp6	*C. obsoletus*	MW633169	–	MW657643	–	–	–	–	–
Csp7	*C. dewulfi*	MW633224	–	MW657644	–	–	–	–	–
Csp21	*C. obsoletus*	MW633234	–	–	–	–	–	–	+
Csp25	*C. obsoletus*	MW633531	–	–	MW657645	–	–	–	–
Csp39	*C. pulicaris*	MW633238	–	–	–	–	–	–	+
Csp112	*C. obsoletus/C. scoticus*	Morphology	–	–	–	MW657646	–	–	–
**Horňa**									
Csp4	*C. obsoletus/C. scoticus*	Morphology	MW657647	–	–	–	–	–	–
Csp26	*C. pulicaris*	MW633240	–	–	–	–	–	–	+
Csp27	*C. obsoletus*	MW633530	MW657648	–	–	–	–	–	–
Csp101	*C. lupicaris*	MW633281	MW657649	–	–	–	–	–	–
Csp102	*C. newsteadi*	MW633283	MW657650	–	–	–	–	–	–
Csp103	*C. obsoletus/C. scoticus*	Morphology	MW657651	–	–	–	–	–	–
Csp104	*C. obsoletus*	MW633284	MW657652	–	–	–	–	–	–
Csp105	*C. obsoletus/C. scoticus*	Morphology	MW657653	–	–	–	–	–	–
**Ostrov**									
Csp5	*C. obsoletus*	MW633523	MW657654	–	–	–	–	–	–
Csp10	*C. pulicaris*	Morphology	MW657655	–	–	–	–	–	–
Csp22	*C. punctatus*	MW633527	MW657656	–	–	–	–	–	–
Csp23	*C. obsoletus*	MW633838	MW657657	–	–	–	–	–	–
Csp28	*C. newsteadi*	MW645480	MW657658	–	–	–	–	–	–
Csp29	*C. newsteadi*	MW633854	MW657659	–	–	–	–	–	–
Csp30	*C. newsteadi*	MW645479	MW657660	–	–	–	–	–	–
Csp31	*C. pulicaris*	MW645471	MW657661	*–*	*–*	*–*	–	–	–
Csp32	*C. pulicaris*	MW638132	–	–	–	–	–	–	+
Csp33	*C. punctatus*	MW639904	MW657662	*–*	*–*	*–*	–	–	–
Csp34	*C. punctatus*	MW645478	MW657663	*–*	*–*	*–*	–	–	–
Csp35	*C. obsoletus*	MW645472	*–*	*–*	*–*	*–*	MW657664	–	–
Csp36	*C. obsoletus*	MW642204	MW657665	*–*	*–*	*–*	–	–	–
Csp37	*C. obsoletus*	MW642205	MW657666	*–*	*–*	*–*	–	–	–
Csp38	*C. obsoletus*	MW642206	MW657667	*–*	*–*	*–*	–	–	–
Csp40	*C. punctatus*	Morphology	–	–	–	–	–	MW657668	–
Csp46	*C. obsoletus*	MW642408	MW657669	*–*	*–*	*–*	–	–	–
Csp47	*C. obsoletus*	Morphology	MW657670	*–*	*–*	*–*	–	–	–
Csp90	*C. riethi*	MW642439	MW657671	*–*	*–*	*–*	–	–	–
Csp92	*C. obsoletus/C. scoticus*	Morphology	MW657672	–	–	–	–	–	–
**Turňa nad Bodovou**									
Csp8	*C. obsoletus*	MW642440	*–*	*–*	–	–	–	MW657673	–
Csp9	*C. pulicaris*	MW642441	MW657674	–	–	–	–	–	–
Csp24	*C. obsoletus/C. scoticus*	Morphology	MW657675	–	–	–	–	–	–
Csp41	*C. lupicaris*	MW642442	MW657676	–	–	–	–	–	–
Csp49	*C. obsoletus*	MW642460	MW657677	*–*	*–*	*–*	*–*	*–*	*–*
Csp50	*C. obsoletus*	MW642474	MW657678	–	–	–	–	–	–
Csp52	*C. newsteadi*	MW642475	MW657679	*–*	*–*	*–*	*–*	*–*	*–*
Csp53	*C. newsteadi*	MW642480	MW657680	*–*	*–*	*–*	*–*	*–*	*–*
Csp54	*C. newsteadi*	MW642500	MW657681	*–*	*–*	*–*	*–*	*–*	*–*
Csp55	*C. punctatus*	MW642502	MW657682	*–*	*–*	*–*	*–*	*–*	*–*
Csp58	*C. pulicaris*	MW642501	MW657683	*–*	*–*	*–*	*–*	*–*	*–*
Csp62	*C. obsoletus/C. scoticus*	Morphology	MW657684	*–*	*–*	*–*	*–*	*–*	*–*
Csp63	*C. obsoletus/C. scoticus*	Morphology	MW657685	–	–	–	–	–	–
Csp64	*C. newsteadi*	MW642503	MW657686	*–*	*–*	*–*	*–*	*–*	*–*
Csp67	*C. obsoletus/C. scoticus*	Morphology	MW657687	*–*	*–*	*–*	*–*	*–*	*–*
Csp68	*C. obsoletus/C. scoticus*	Morphology	MW657688	*–*	*–*	*–*	*–*	*–*	*–*
Csp71	*C. furcillatus*	Morphology	MW657689	–	–	–	–	–	–

+ Positive, − Negative.

The PCR reactions were performed in 50 μL of the reaction mixture consisting of: 1.5 μL of total genomic DNA template; 25 μL of OneTaq^®^2x Master Mix with Standard Buffer; 21.5 μL of nuclease free water; and 1 μL of each primer (Sigma-Aldrich). The reactions were carried out in a programmable Master cycler nexus X2 (Eppendorf, Germany) using an amplification program consisting of an initial denaturing step at 95 °C for 5 s, 5 cycles (94 °C, 40 s; 45 °C, 40 s; 72 °C, 60 s), followed by 45 cycles (94 °C, 40 s; 50 °C, 40 s; 72 °C, 1 s), and a final elongation at 72 °C for 7 min [29].

### Blood-meal molecular analysis

Molecular identification of the blood meals from individual blood-engorged females was performed by sequencing part of the cytochrome b (cyt b) mitochondrial gene (350 bp). All PCRs were performed in a total volume of 47 μL, including 3 μL of total genomic DNA template, 21 μL of Emerald AmpGT PCR Master Mix (Takara, Shiga, Japan), 21 μL of nuclease-free water, and 1 μL of each primer (Sigma-Aldrich). The PCRs were performed on a Master cycler nexus X2 (Eppendorf, Germany) using universal vertebrate primers, forward cyt bb1 (5′–CCA TCM AAC ATY TCA DCA TGA AA–3′) and reverse cyt bb2 (5′–GCH CCT CAG AAT GAY ATT TGK CCT CA–3′) [35]. The amplification process consisted of an initial denaturation step at 94 °C for 5 min, then 35 × (94 °C for 1 min, 55 °C for 1 min, 72 °C for 1 min), and elongation at 72 °C for 7 min.

The following steps including visualization, purification, and sequencing of PCR products were the same for the determination of *Culicoides* spp., as well as for host blood diagnostics, i.e., 5 μL of amplicons were run on 1% agarose gel, stained with GoodView^TM^ Nucleic Acid Stain and visualized under UV light. The PCR products were sent to the Microsynth Seqlab (Vienna, Austria) or SEQme (Dobříš, Czech Republic) for purification and sequencing. The sequencing was performed in both strands with identical primers used for the PCR.

### Data analyses

The sequences of both COI and cyt b genes represented by chromatograms were analyzed and edited using MEGA X software [22]. Assemblage of the nucleotide sequences was carried out in Gene Tool Lite 1.0 software (BioTools Inc., Jupiter, FL, USA). The sequences of COI or cyt b genes obtained by sequencing in our study were compared to the reference sequences from the NCBI database using Blast (Basic Local Alignment Search Tool). When the similarity of our COI or cyt b gene sequences reached ≥98%, the corresponding taxonomic identity was assigned to the reference sequence.

## Results

A total of 28,752 individuals of 17 species of *Culicoides* biting midges were captured on the cattle and horse farms (Table 2). The highest abundance was observed in *C. obsoletus/C. scoticus* species *n* = 26,137 (90.9%). Biting midges from the Culicoides subgenus represented 7.4% (*n* = 2137) of the total number of biting midges. Individual percentages were as follows: *C. newsteadi* Austen, 1921 *n* = 1112 (3.9%); *C. punctatus* Meigen, 1804 *n* = 595 (2.1%); *C. lupicaris* Downes & Kettle, 1952 *n* = 320 (1.1%); *C. pulicaris* Linnaeus, 1758 *n* = 97 (0.3%); and *C. bysta* Sarvašová & Mathieu, 2017 *n* = 13 (0.05%). Other biting midges included *C. furcillatus* Callot, Kremer & Paradis, 1962 *n* = 229 (0.8%); *C. festivipennis* Kieffer, 1914 *n* = 72 (0.3%); *C. riethi* Kieffer, 1914 *n* = 33 (0.1%); *C. circumscriptus* Kieffer, 1918 *n* = 33 (0.1%); and *C. stigma* Meigen, 1818 *n* = 28 (0.1%). Less than 10 captured individuals were of *C. slovacus* Országh, 1969*/C. tauricus* Gutsevich, 1959, *C. salinarius* Kieffer, 1914, *C. clastieri* Callot, Kremer & Deduit, 1962, *C. subfascipennis* Kieffer, 1919, and *C. dewulfi* Goetghebuer, 1936 species*.* Of 28,696 females, 54% (15,516) were nulliparous, 9486 (31.1%) were parous, 10.8% (3102) were classified as fully engorged, and 2.1% (592) were gravid (Table 2).

**Table 2:** Species and abundance of *Culicoides* females trapped on four farms.

Species	Košice (% total)	Turňa nad Bodvou (% total)	Ostrov (% total)	Horňa (% total)	Total (% total)
*C. obsoletus/C. scoticus*	20,154 (98.1)	4523 (87.4)	1085 (42.3)	375 (80.5)	26,137 (90.9)
*C. punctatus*	57 (0.3)	70 (1.4)	453 (17.7)	15 (3.2)	595 (2.1)
*C. lupicaris*	144 (0.7)	65 (1.3)	60 (2.3)	51 (10.9)	320 (1.1)
*C. pulicaris*	15 (0.07)	70 (1.4)	3 (0.1)	9 (1.9)	97 (0.3)
*C. newsteadi*	29 (0.1)	217 (4.2)	851 (33.2)	15 (3.2)	1112 (3.9)
*C. bysta*	4 (0.02)	7 (0.1)	2 (0.08)	–	13 (0.05)
*C. furcillatus*	103 (0.5)	123 (2.4)	1 (0.04)	–	229 (0.8)
*C. festivipennis*	4 (0.02)	6 (0.1)	62 (2.4)	–	72 (0.3)
*C. circumscriptus*	5 (0.02)	6 (0.1)	22 (0.9)	–	33 (0.1)
*C. delta*	5 (0.02)	–	–	–	5 (0.02)
*C. stigma*	8 (0.04)	17 (0.3)	3 (0.1)	–	28 (0.1)
*C. riethi*	–	32 (0.6)	–	1 (0.2)	33 (0.1)
*C. subfascipennis*	–	1 (0.02)	–	–	1 (0.003)
*C. slovacus/C. tauricus*	–	3 (0.06)	6 (0.2)	–	9 (0.03)
*C. salinarius*	–	–	8 (0.3)	–	8 (0.03)
*C. clastieri*	–	–	1 (0.04)	–	1 (0.003)
*C. dewulfi*	1 (0.005)	–	–	–	1 (0.003)
*Culicoides* spp. (unidentified)	–	2 (0.04)	2 (0.08)	–	4 (0.01)
*Culicoides* ***–*** males	21 (0.1)	31 (0.6)	4 (0.2)	–	56 (0.2)
*Culicoides* **–** females	20,529	5142	2559	466	28,696
Females’ physiological status					
Nulliparous	12,096 (58.9)	2073 (40.4)	1104 (43.1)	243 (52.1)	15,516 (54.0)
Parous	5468 (26.7)	2588 (50.3)	1236 (48.4)	194 (41.6)	9486 (33.1)
Engorged	2834 (13.8)	151 (2.9)	113 (4.4)	4 (0.9)	3102 (10.8)
Gravid	131 (13.8)	330 (6.4)	106 (4.1)	25 (5.4)	592 (2.1)
Total	20,550	5173	2563	466	28,752

The samples also included males (*n* = 56; 0.2%) that were recorded in three localities. Twenty-one males of *C. obsoletus*/*C. scoticus* were trapped in the riding area of Košice. We also recorded 33 males, namely *C. obsoletus*/*C. scoticus* (*n* = 32) and *C. punctatus* (*n* = 1) on a farm in Turňa nad Bodvou. One piece of *C. obsoletus*/*C. scoticus* and *C. lupicaris* were obtained from a farm in Ostrov.

Detection of the host’s blood was carried out with 48 samples of biting midges of *C. obsoletus/C. scoticus*, *C. dewulfi*, *C. punctatus*, *C. pulicaris*, *C. newsteadi*, *C. lupicaris*, *C. tauricus/slovacus*, *C. riethi*, and *C. furcillatus* species*.* We detected host blood from species of biting midges that had an abdomen filled with blood (not all species of trapped *Culicoides* in our study had blood in the abdomen). We selected representative samples. The majority of engorged females were from *C. obsoletus*/*C. scoticus*. We randomly selected females and tried to diagnose engorged females from each locality for host blood meal analysis. It was observed that altogether seven species of biting midges took blood from the cattle (*Bos taurus*), in particular: *C. obsoletus/C. scoticus*, *C. punctatus*, *C. pulicaris*, *C. newsteadi*, *C. lupicaris*, *C. riethi*, and *C. furcillatus.* Horse blood (*Equus caballus*) was detected in the females of *C. dewulfi* species. Humans were parasitised by *C. punctatus* and *C. obsoletus*. The European hare (*Lepus europaeus*) and chicken (*Gallus gallus*) were fed on by the biting midges of the *C. obsoletus* species. The blood of the yellow-necked mouse (*Apodemus flavicollis*) was diagnosed with *C. obsoletus/C. scoticus* (Table 1).

## Discussion

Determining species composition and detecting host blood are essential to control the spread of new species of biting midges, and the consequent transmission of pathogens. Thanks to the monitoring of *Culicoides* species, we can prevent the introduction of new pathogens into areas where they have not occurred before. Our work was the first study to investigate the mitochondrial gene for cytochrome b of vertebrae in Slovakia. This gene facilitates the identification of hosts parasitized by biting midges. Most species of *Culicoides* biting midges parasitize birds or mammals; certain species, however, feed on the blood of reptiles and amphibians [9]. Multiple studies investigating the hosts of biting midges, for example, in Denmark [24], presented 8 species of mammals and 7 species of birds as the most frequent hosts. For *Culicoides* spp., 9 mammalian and 24 avian species were detected as host for Romania [45]. In Germany, Santiago-Alarcon et al. [39] monitored the hosts of biting midges in forests located near human dwellings. In the Czech Republic, Votýpka et al. [49] examined biting midges parasitizing birds. In Great Britain, they examined the parasite–host relationships between biting midges and exotic animals kept in zoological gardens [16]. These data contributed to the knowledge that hematophagous females of biting midges parasitize a wide range of vertebrates, but they prefer mammals and birds [26]. For example, *C. kibunensis* parasitize birds and *C. chiopterus* and *C. deltus* preferably parasitize mammals. The majority of species of biting midges, such as *C. obsoletus* and *C. festivipennis*, belong to non-host specific species, which take blood from both mammals and birds [39].

Although biting midges had been paid less attention than other blood-sucking vectors in the past, the interest in these Diptera has increased enormously over the last two decades, mainly due to the economic losses suffered by ruminant breeders which resulted from viral transmission (BTV, SBV) [44]. The present research was carried out on four cattle and horse farms in Eastern Slovakia, with the result of the confirmed presence of 17 species of biting midges. Given that host blood can only be collected from blood-engorged females, the females of 10 species of biting midges were selected and the hosts they parasitized were identified. In addition to the detection of the host’s blood, each sample was also examined using the partial mitochondrial cytochrome c oxidase subunit I gene (COI), and this facilitated the molecular confirmation of the species of biting midges, in addition to the morphological diagnostics.

The highest abundance of females with blood was observed in biting midges of the Culicoides subgenus, which parasitized hosts in 6 groups. Individuals collected within our study represented the *C. obsoletus/C. scoticus* and *C. dewulfi* species. *Culicoides obsoletus/C. scoticus* species belong to the most widespread species present in the Palaearctic realm, and they prefer mammals as their hosts. However, 14% of studies confirmed avian hemosporidian parasites in parous females of *Culicoides obsoletus/C. scoticus* [6]. It was impossible to distinguish between *C. obsoletus* and *C. scoticus* based on morphological features; this is why a PCR analysis was carried out with the aim of confirming the species [1]. In our study, molecular methods were applied to confirm *C. obsoletus*; out of 27 examined samples, 74% parasitized cattle (*Bos taurus*). Our results are similar to those of other studies [4, 30, 39], which concluded that cattle were the most frequent species on which *Culicoides* biting midges fed in Europe. *Culicoides obsoletus* parasitizing cattle were detected on each of the monitored farms. As the samples were also collected near horses, it was obvious that another identified host of these biting midges was horses (*Equus caballus*).

The light traps used to collect the biting midges were installed as usual, i.e., at the height of approximately 1.5 m above the ground. However, scientists who have examined biting midges parasitizing birds claim that due to the behavior of avian hosts, it is advisable to place the light traps at the height of four meters [8, 49]. As our traps were placed at lower heights, the captured biting midges included those parasitizing small mammals; in particular, the yellow-necked mouse (*Apodemus flavicollis*) and the European hare (*Lepus europaeus*). As for the avian hosts, one species was detected, i.e., a chicken (*Gallus gallus*). The chicken was parasitized by biting midges of the *C. obsoletus* species in the horse-riding arena where no poultry was kept. However, on the other side of the horse-riding arena, there were family houses with gardens where the poultry were bred. Biting midges are most abundant in proximity to production breeding sites. They disperse into surrounding areas in search suitable hosts. The flight distance for any individual, however, can be much lower or higher [21, 42]. Actually, *Culicoides* biting midges are not good fliers; they are only able to fly to the maximum distance of 5 km within several days. With strong winds, however, they may travel more than 100 km [30]. We assume that it was particularly the airflow that facilitated the detection of biting midges with the chicken blood inside the horse-riding arena. Host availability has a significant impact on the observed host-feeding patterns of *Culicoides* spp. that can also be used to estimate dispersal distances [21, 31]. *Culicoides* feed on reptiles, birds, mammals, and humans. Females must locate on the host in order to draw blood, and this process is influenced and controlled to some extent by environmental factors such as sunlight, wind speed, humidity, and ambient temperature, which can limit flight activity [7]. The daily rhythm of the attack is influenced by a periodically repeating change in temperature and light. Most midges are active at the dusk and at night, but some species are active during the day, with an increase in activity observed in the morning after sunrise and in the afternoon around sunset. *Culicoides obsoletus* attack the host during the day. Females prefer to feed on the host in warm humid and windless weather. Flight activity conditioned by host search is affected by temperature, humidity, and light intensity and inhibited by wind. Culicoides also fly to altitudes 170–200 m above the ground, where the temperature inversion peaks during the early hours of the night. Local maxima of air temperature and wind speed at this altitude are particularly favorable for insect migration, as showed by Chapman et al. [12], Reynolds et al. [37], and Sanders et al. [38], who trapped 14 species at these altitudes, including *C pulicaris*, *C. punctatus*, *C. nubeculosus*, and species from the subgenus Avaritia – *C. obsoletus*, *C. scoticus*, *C. dewulfi*, and *C. chiopterus*. According to a study by Kluisters et al. [21] in the United Kingdom, Obsoletus Group members successfully disperse between farms. Similarly, Mignotte et al. [31] confirmed the high dispersing capacity of *C. obsoletus* in France [32]. They confirmed that species of the Obsoletus Group are able to disperse 2.5 km or more, with males able to disperse to a distance of 1 km in 24 h.

Biting midges of the Culicoides subgenus include 14 species, i.e., *C. pulicaris*; *C. lupicaris*; *C. impunctatus*; *C. punctatus*; *C. grisescens*; *C. newsteadi*; *C. flavipulicaris*; *C. fagineus*; *C. subfagineus*; *C. bysta*; *C. paradoxalis.*; *C. boyi*; *C. selandicus*; and *C. kalix* [51]. The faunistic entomological papers published in Slovakia [33, 41, 43, 47] reported that the Culicoides subgenus contained the following 5 species of biting midges: *C. pulicaris*, *C. punctatus*, *C. newsteadi*, *C. lupicaris*, and *C. bysta*. As for the Culicoides subgenus, we examined 22 samples of biting midges, i.e., *C. newsteadi* (*n* = 8), *C. pulicaris* (*n* = 7), *C. punctatus* (*n* = 5), and *C. lupicaris* (*n* = 2). We confirmed again that cattle (*Bos taurus*) were the main host species. However, *C. punctatus* species held the second place in our study, and these individuals were confirmed to contain human blood in their abdomens. Similarly, data reported from Bulgaria indicate that *C. punctatus* is a source of human host blood [8]. It was also observed in this study that 4 other species of biting midges parasitized cattle, including *C. riethi*, a species that occurs only rarely. Similar results were reported by Martínez-de la Puente et al. [27] who confirmed in their study that these biting midges contained bovine blood.

In our study, diagnosis of a physiological condition based on morphological features was not always accurate [15] because it was not possible to successfully diagnose all engorged females of biting midges. Even though their abdomens were red, this did not mean they contained blood. They might have been filled with other liquids, such as herbal juices for example. Moreover, females of different species may have abdomens of different colors, and this may cause a negative result of a PCR analysis. Furthermore, blood contained in females might have degraded while being stored in ethanol [32], or biting midges might have taken blood from animals with sequences not yet included in GenBank. Females might also have taken blood from invertebrates with hemolymph circulating in their bodies. As for our results, out of the 48 examined samples, in 4 cases (8%) a host could not be identifiable.

The blood taken by biting midges affects their intestinal microflora and this is also a source of proteins, which females needed to lay eggs. Some studies confirmed that the selection of a suitable host for *Culicoides* biting midges may have been limited by the host availability in the area where they live [34]. On the other hand, anthropogenic activity facilitates the spread of vectors, as well as new pathogens to people. These implications were examined in Germany [39], where it was assumed that people who live near forests are exposed to a wide range of parasites of wild animals. Vectors that feed on various hosts probably change the source of their food when environmental conditions change, and thus a change between the vector and the host occurs. This means that the species taking blood of mammals parasitize birds, and vice versa [48]. The selection of a suitable host, blood ingestion, and transmission of pathogens are also affected by other factors, including the biting midges count, the presence of a host, and the frequency of feeding. All these factors affect the transmission of pathogens [18, 20]. Considering our results, we assume that potential vectors of pathogens, i.e., biting midges, parasitize a wide range of hosts. Further research is required, particularly on hosts that are only parasitized by certain specific species of biting midges. Such hosts are, for example, horses kept in the horse-riding arena subjected to our monitoring.

## Conclusions

Our study was the first of its kind in Slovakia, and it was aimed at identifying a diversified range of hosts from which biting midges took blood. The dominant species found at the chosen locations was *Culicoides obsoletus*, representing 91.1% of the fauna out of all captured biting midges. Particularly *C. obsoletus* was designated as a potential vector of BTV and SBV [17, 23]. Our results indicate that biting midges select their hosts opportunistically, preferably cattle; this piece of information should be taken into account in epizootiological model studies. Bartsch et al. [4] and Ayllón et al. [2] made the same conclusions in their studies. The results of this study may be used in the future as an important indicator when modelling the transmission of pathogens and designing veterinary emergency plans.

## Conflict of interest

The authors declare that they have no conflict of interest.

## Funding and source

This research was funded by project VEGA No. 1/0043/19 (Share 0.7), and project “Open scientific community for modern interdisciplinary research in medicine (OPENMED)”, ITMS2014+: 313011V455 supported by the Operational Programme Integrated Infrastructure, funded by the ERDF (Share 0.3).

## Author contribution

Conceived the study: ZK, AK; Designed the experiment: ZK, AK, AS; Performed the field activities and sampling: ZK, AK; Performed the laboratory work: ZK, AKI, AS; Analyzed and interpreted the data: ZK, AS, AKI; Wrote the original draft of the manuscript: AK, AS, AKI; Reviewed and edited the final version of the manuscript: AK, ZK, AS; Supervision: AK.
